# Cloves Regulate Na^+^-K^+^-ATPase to Exert Antioxidant Effect and Inhibit UVB Light-Induced Skin Damage in Mice

**DOI:** 10.1155/2021/5197919

**Published:** 2021-08-21

**Authors:** Xi Gao, Fuling Luo, Hengguang Zhao

**Affiliations:** ^1^Department of Dermatology, University-Town Hospital of Chongqing Medical University, Chongqing 401331, China; ^2^Medical Sciences Research Center, University-Town Hospital of Chongqing Medical University, Chongqing 401331, China; ^3^Department of Pharmacy, The First Affiliated Hospital of Chongqing Medical University, Chongqing 400016, China

## Abstract

The purpose of this study was to observe the effect of cloves (*Syzygium aromaticum* (L.) Merr. & L.M. Perry) on the mouse skin using a UVB-induced skin injury mouse model. The serum, liver, and skin indexes of mice were determined by kits, H&E tissue staining, and qPCR assay. The compound composition of cloves was determined by HPLC. The results showed that cloves increased the activity of Na^+^-K^+^-ATPase in the skin and then maintained the sodium and potassium pump in the damaged skin muscle membrane. Cloves alleviated the oxidative stress injury induced by UVB irradiation by normalizing the related oxidative stress indexes (T-SOD, CAT, AGEs, and H_2_O_2_) in serum and skin. Inhibition of the proinflammatory cytokines TNF-*α*, IL-1*β*, and IL-6 and increased activation of anti-inflammatory cytokines IL-4 and IL-10 occurred after treatment with cloves, which ultimately reduced the inflammatory damage to the body. Further results showed that cloves upregulate SOD1, SOD2, CAT, GSH, IL-10, I*κ*B-*α*, AMPK, SIRT1, LKB1, PGC-1*α*, APPL1, and FoxO1 and downregulate NF-*κ*B p65, TNF-*α*, IL-6, and mTOR mRNA expression in the skin tissues of UVB-damaged mice. The results of composition analysis showed that the five most abundant compounds in cloves are rutin, isoquercitrin, ferulic acid, dihydroquercetin, and quercitrin. Cloves regulate the skin sarcomembrane Na^+^-K^+^-ATPase through these five compounds, and because they regulate the oxidation, inflammation, and ATP energy consumption of the body, they subsequently protect the skin from UVB damage.

## 1. Introduction

The skin is the first barrier between the human body and the outside environment and plays many roles such as protecting the body and regulating body temperature, sensation, secretion, excretion, and immunity. Skin damage caused by various external stimuli mainly includes physical and chemical damage. Ultraviolet (UV) light, infrared (IR) light, dust, and haze will cause physical damage, among which UV radiation is the most significant [1]. The acute skin injury caused by UV radiation mainly manifests as a red and dry cortex, damaged epidermis, muscle relaxation, rough folds, local abnormal pigment deposition, and leather-like appearance. Studies have shown that damage by light can lead to severe keratinization of the skin that prevents the transfer of substances and energy. It is mainly manifested as a flattening of the epidermal layer, disappearance of the epidermal protrusion and papillary layer, distortion of the epidermal capillaries, decreased immune function, and inflammatory proliferation of melanoma cells [2].

The pathological features of the damaged skin tissue are mainly the disintegration of collagen fibers of the extracellular matrix (ECM), loss of collagen, and infiltration of inflammatory cells. At present, people are increasingly concerned about skin light damage, which not only affects human health but also brings physical and mental stress to people, affects beauty, and even causes mental diseases [3].

Cloves are the flower buds harvested from a tree (*Syzygium aromaticum* (L.) Merr. & L.M. Perry) in the Myrtaceae family. They are used in Chinese medicine or functional food and worldwide and have antimicrobial and immune-enhancing properties [4, 5]. In industry, cloves are also used to make cosmetics [6]. Studies have shown that cloves can effectively accelerate skin wound healing and reduce inflammation and scarring. Therefore, there may be value in using cloves to inhibit photodamage to the skin [7].

In laboratory studies, animal models of skin photodamage are often used. UVB (280-320 nm) is the most harmful band for organisms, and therefore, animal skin photodamage models are often modeled with UVB, and the skin tissue changes caused by this modeling method are very similar to those that result after photodamage to the human skin [8]. The current study also used this method to establish an animal model and observe the inhibitory effect of cloves on UVB-induced skin injury.

Na^+^-K^+^-ATPase, also known as the sodium-potassium pump, is a membrane transporter. It participates in the dual activities of carrier and enzyme and is embedded in the cell membrane. It can hydrolyze ATP into ADP and phosphoric acid, which subsequently releases energy. The ATPase activity level directly reflects changes in the mitochondrial function. When Na^+^-K^+^-ATPase activity is reduced, the mitochondrial sodium pump function is impaired, leading to edema of the mitochondrial intima and consequent decrease in mitochondrial membrane fluidity [9]. Free radicals have the ability to damage Na^+^-K^+^-ATPase. This results in a decrease in membrane fluidity, which leads to the impairment of mitochondrial oxidative phosphorylation, while a deficiency in ATP synthesis can directly decrease the activity of Na^+^-K^+^-ATPase [10].

Studies have found that when the exogenous skin tissue is damaged, injury-stimulating factors will initiate the process of inflammation, repair, and remodeling, which eventually leads to wound healing. After tissue cells are damaged, they will release a variety of endogenous substances with immunomodulatory activity, namely, damage-associated molecular patterns (DAMPs), which participate in the synergistic activation of the innate immune system and the regulation of acquired immune direction and regulate tissue repair or abnormal remodeling. It should be noted that ATP is not only the main energy substance for maintaining cellular metabolic function under physiological states but it also can be used as a DAMP to regulate various cellular effects under stress states [11–13].

ATP metabolism and purine signal levels are low in the inflammatory reaction stage of diabetic wound healing. Exogenous ATP supplementation accelerates the healing of diabetic wounds. It is further suggested that extracellular ATP metabolism is related to the process of inflammatory wound repair, and amplification of extracellular ATP signals in the early stage of acute injury can promote diabetic wound healing [14]. This indicates that a normal Na^+^-K^+^-ATPase level is an important factor for maintaining normal skin shape or promoting skin self-repair when the skin is exposed to external stimulation [15]. However, there are few studies on Na^+^-K^+^-ATPase in the skin at present. In this study, we will further observe the mechanism used by cloves to inhibit UVB-induced skin injury, which involves repairing the damaged skin sarcomembrane Na^+^-K^+^-ATPase, so as to develop a theoretical basis for the use of cloves as a skin-protective active substance with a clear mechanism.

## 2. Materials and Methods

### 2.1. Extraction of Cloves

Extraction of 500 g of dried cloves (Quanzhou Bainian Xiuben Tea Industry Co., Ltd, Quanzhou, Fujian, China) was performed twice with 10 : 1 (w/v) ethanol, for 2 h each time. The two extracts were combined and then dried in a rotary evaporator (R1020, Zhengzhou Greatwall Scientific Industrial and Trade Co., Ltd., Zhengzhou, Henan, China) to obtain the clove extract.

### 2.2. Composition Analysis Using HPLC

The clove extract and a standard substance were dissolved in methanol, and the solution was transferred to brown glass liquid vials for measurement after being filtered through the 0.22 *μ*m membrane filter. The compounds in cloves were determined by HPLC (UltiMate3000 HPLC system, Thermo Fisher Scientific, Waltham, MA, USA) using the Accucore C18 column (column packing size of 5 *μ*m, column diameter of 4.6 mm, column length of 250 mm) under the following chromatographic conditions: mobile phase for A of 0.5% acetic acid in water and B of acetonitrile, flow rate of 0.5 mL/min, column temperature at 30°C, detection wavelength at 359 nm, injection volume of 5 *μ*L, and preequilibrium of 10 min. The mobile phase is displayed as Table 1.

### 2.3. Experiments with Mice

Fifty female ICR mice (7 weeks old) were obtained from the Experimental Animal Center of Chongqing Medical University (Chongqing, China, SCXK (Yu) 2018-0003). The mice were placed at a temperature of 25 ± 2°C and a relative humidity of 50 ± 5%, and the light/dark cycle was performed for 12 h. The mice could eat standard food and drinking water freely. The mice were randomly divided into 5 groups, and each group had 10 mice: (i) normal group, (ii) UVB group (model group), (iii) VC + UVB group, (iv) low concentration of cloves+UVB (clove-L + UVB) group, and (v) high concentration of cloves+UVB (clove-H + UVB) group. At the beginning of the experiment, food and water were provided ad libitum for the mice in the normal group and the UVB group for 14 days. Vitamin C by gavage at the daily intake of 300 mg/kg was treated to the mice in the VC + UVB group. Clove extract was administered by gavage at 150 and 300 mg/kg/day in the clove-L + UVB group and clove-H + UVB group.

From day 15 to day 28, a patch of hair approximately 2 cm^2^ in size on the backs of the mice was shaved with an electric razor. Then, the mouse skin injury model was established in the other groups (with the exception of the normal group) using ultraviolet radiation equipment, and mice were irradiated for 2 h every day. The VC + UVB group, clove-L + UVB group, and clove-H + UVB group continued to receive samples by gavage according to the action pattern of the previous 14 days. On the 29th day, all mice were euthanized, and tissue and whole blood were collected for experiments.

### 2.4. Hematoxylin and Eosin (H&E) Tissue Staining

After euthanization, 0.5 cm × 0.5 cm pieces of liver tissue and hairless skin on the backs of the mice were excised. The tissues were fixed in 4% paraformaldehyde solution (Fuzhou Phygene Biotechnology Co., Ltd, Fuzhou, Fujian, China), and then H&E (Fuzhou Phygene Biotechnology Co., Ltd.) staining was performed. Finally, the pathological morphology of the mice tissue was photographed using the upright microscope (BX43, Olympus, Tokyo, Japan).

### 2.5. Kit Test Indexes

The serum and skin tissue IL-4, IL-6, IL-10, TNF-*α*, IL-1*β*, T-SOD, CAT, AGEs, H_2_O_2_, Na^+^-K^+^-ATPase, and NAD^+^ levels in mice were analyzed using the test kits (Shanghai Enzyme-linked Biotechnology Limited Company, Shanghai, China).

## 3. qPCR Assay

The mouse skin tissue was homogenized, and total RNA was extracted with TRIzol Reagent (Beijing Solarbio Biotechnology Co., Ltd., Beijing, China). The RNA was then reverse transcribed into cDNA using the kit (Thermo Fisher Scientific). The mixed system liquid (cDNA: 1 *μ*L; TaqMan™ Multiplex Master Mix: 10 *μ*L, Thermo Fisher Scientific; primer: 10 *μ*M, 2 *μ*L, Thermo Fisher Scientific, Table 2 [16]; ddH_2_O: 7 *μ*L, Beijing Solarbio Biotechnology Co., Ltd.) was placed in the PCR instrument (95°C for 15 s, 55°C for 30 s, and then 72°C for 35 s by 40 cycles, Stepone Plus PCR instrument, Thermo Fisher Scientific) for detection. And the 2^-*ΔΔ*CT^ method was using for calculating the relative strength of the expression. *β*-Actin was used as internal reference gene for this experiment [17].

### 3.1. Statistical Analysis

The experimental data of this study were analyzed by SPSS 17.0 and GraphPad Prism 7 statistical software. The mean ± standard deviation (SD) represents the results of the experimental data. The differences between the experimental data of each group were analyzed by one-way ANOVA and *t*-test (*P* < 0.05), and all analyses were performed three times.

## 4. Results

### 4.1. Constituents in Cloves

**Figure 1:**
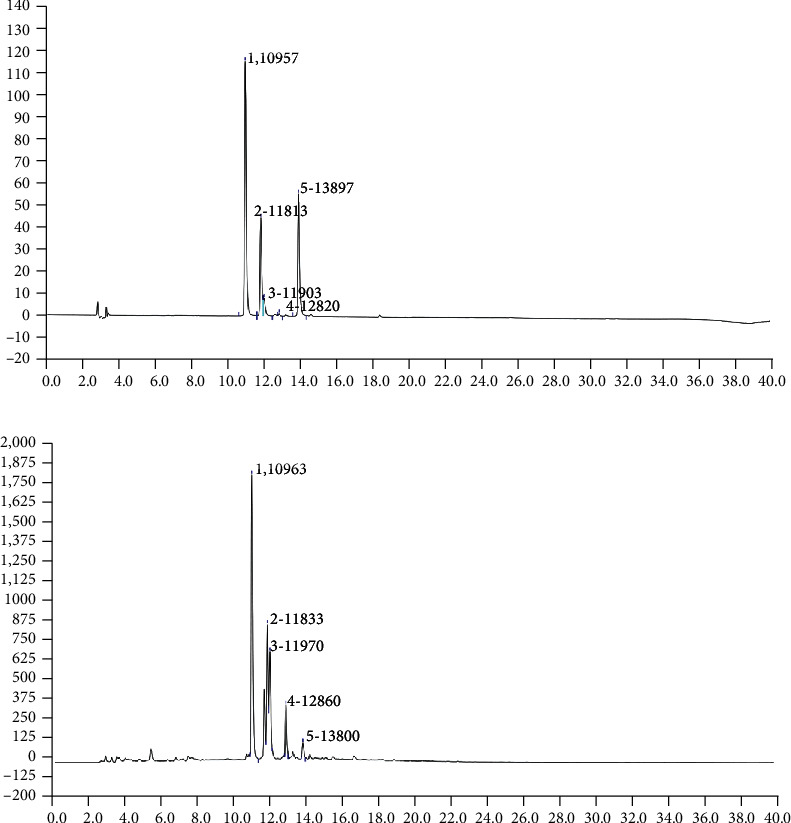
HPLC chromatogram of standard substance (a) and clove constituents (b). 1: rutin; 2: isoquercitrin; 3: ferulic acid; 4: dihydroquercetin; 5: quercitrin.

The clove extract was analyzed by HPLC, and five main compounds were detected (Figure 1), which were rutin, isoquercitrin, ferulic acid, dihydroquercetin, and quercitrin. The peak area of rutin was the largest, which confirmed that of the 5 constituents, the amount of rutin in the extract of cloves was the highest.

### 4.2. Pathological Observation of the Liver and Skin in Mice

**Figure 2:**
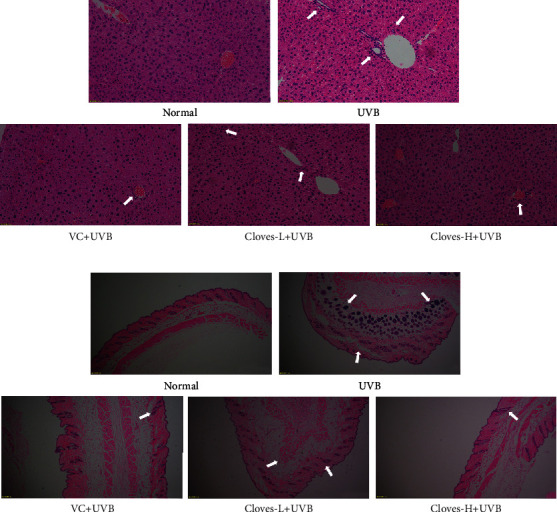
H&E staining pathological observation of the liver (a) and skin (b) in mice.

As shown in Figure 2(a), the liver structure of mice in the normal group was complete. The liver cells were orderly arranged around the central vein in the form of satellite emissions. The nuclei were large and round, with morphological characteristics of normal liver cells, and there was no infiltration of inflammatory cells. Compared with the normal group, the arrangement of liver cells in the UVB group was more disordered, and the liver cells around the central vein exhibited partial necrosis and infiltration of inflammatory cells. When compared to the UVB group, the pathological structures of liver cells in the VC + UVB group, clove-L + UVB group, and clove-H + UVB group were all ameliorated to varying degrees, and the morphology of liver cells in the clove-H + UVB group was similar to that in the normal group.

Figure 2(b) shows that the skin structure of mice in the normal group was complete, with a thin epidermal layer, a wavy junction between epidermis and dermis, and without excessive keratinization of the cuticle. The dermis was thicker, and the collagen bundles were orderly and evenly distributed. In the UVB group, the thickness of the dermis was significantly thinner, the number of collagen fiber bundles was significantly reduced, the subcutaneous tissue was disordered, and the boundary was not obvious. In addition, there was inflammatory cell infiltration around the appendages, which is a sign of the occurrence of a chronic inflammatory reaction in the skin, and indicates that long-term UVB irradiation can induce skin photodamage. Compared with the UVB group, the thickness of the skin dermis was increased in the VC + UVB group, but the collagen fibers were dispersed and loose. The thickness of the skin from mice in the clove-H + UVB group was significantly thicker than that in the UVB, VC + UVB, and the clove-H + UVB groups. The distribution of collagen fiber bundles was relatively uniform and orderly, and the overall structure was similar to that in the normal group.

From day 15 to day 28, a patch of hair approximately 2 cm^2^ in size on the backs of the mice was shaved with an electric razor. Then, the mouse skin injury model was established in the other groups (with the exception of the normal group) using ultraviolet radiation equipment, and mice were irradiated for 2 h every day. The VC + UVB group, Cloves-L + UVB group, and Cloves-H + UVB group continued to receive samples by gavage according to the action pattern of the previous 14 days. On the 29th day, all mice were euthanized, and tissue and whole blood were collected for experiments.

### 4.3. Oxidative Indicators of Skin Injury in Mice

As shown in Table 3, the enzymatic activities of T-SOD and CAT in serum and the skin of mice in the normal group were the highest, and the amounts of H_2_O_2_ and AGEs were the lowest. In contrast, the levels of the above indexes in the UVB group showed a completely opposite trend, and there was a significant difference between the normal group and the UVB group (*P* < 0.05). Compared with the UVB group, the activities of T-SOD and CAT in the serum and skin of mice were significantly increased after vitamin C and clove treatment, with a significant decrease in H_2_O_2_ (*P* < 0.05). In particular, the levels of T-SOD, CAT, H_2_O_2_, and AGEs in the serum and skin of mice in the clove-H + UVB group were the most similar to those in the normal group, indicating that cloves can greatly improve the activity of antioxidant enzymes in the serum and skin of mice, with a stronger effect than that of the common antioxidant substance vitamin C.

### 4.4. Na^+^-K^+^-ATPase and NAD^+^ Levels in the Mouse Skin Tissue

**Figure 3:**
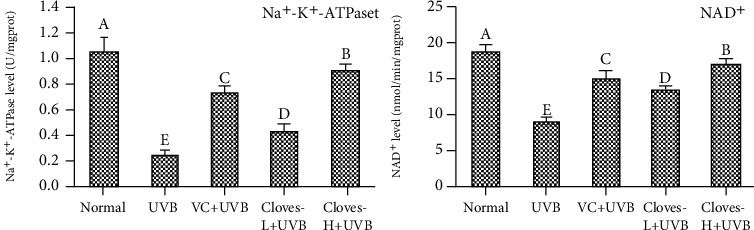
Na^+^-K^+^-ATPase and NAD^+^ levels in the skin tissue of mice.^a–e^Different letters indicate significant differences between the two groups (*P* < 0.05) according to Duncan's honestly significantly different test.

Figure 3 shows that the levels of Na^+^-K^+^-ATPase and NAD^+^ in the skin tissues of mice in the normal group (1.05 U/mgprot and 18.68 nmol/min/mgprot) were the highest, while the above indexes in the UVB group (0.24 U/mgprot and 8.92 nmol/min/mgprot) were the lowest. Compared with the UVB group, the levels of Na^+^-K^+^-ATPase and NAD^+^ in the skin of mice in the VC + UVB group (0.72 U/mgprot and 15.13 nmol/min/mgprot), clove-L + UVB group (0.43 U/mgprot and 13.44 nmol/min/mgprot), and clove-H + UVB group (0.90 U/mgprot and 17.01 nmol/min/mgprot) were all increased to varying degrees. The levels of Na^+^-K^+^-ATPase and NAD^+^ in the skin of mice treated with the high concentration of cloves (clove-H + UVB group) were not significantly different from those in the normal group.

### 4.5. Inflammatory Indicators of Skin Injury in Mice

Table 4 shows that the levels of proinflammatory cytokines TNF-*α*, IL-1*β*, and IL-6 in the serum and skin of mice in the UVB group were the highest, while the levels of anti-inflammatory cytokines IL-4 and IL-10 were the lowest, and the UVB group exhibited an opposite trend compared with the normal group. Compared with the UVB group, after treatment with VC and cloves, the proinflammatory cytokines in the serum and skin of mice were decreased to varying degrees, while the anti-inflammatory cytokines were increased. There was a greater effect from clove-H + UVB, and it resulted in greater normalization of inflammatory indexes as compared to those of VC + UVB and clove-L + UVB, and the results were similar to those of the normal group.

## 5. mRNA Expression in Mouse Skin Tissues

**Figure 4:**
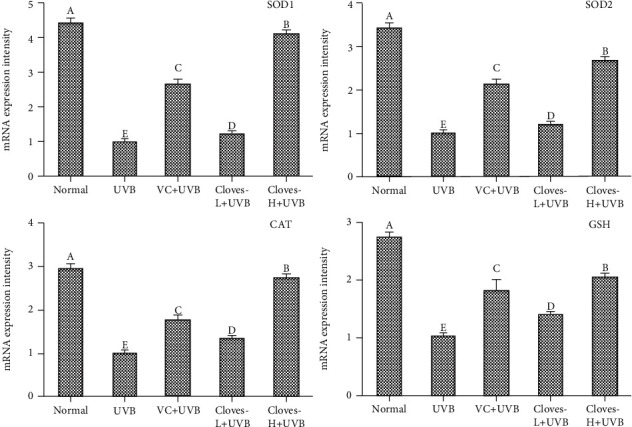
mRNA expression levels of SOD1, SOD2, CAT, and GSH in the skin tissue of mice.^a–e^Different letters indicate significant differences between the two groups (*P* < 0.05) according to Duncan's honestly significantly different test.

As shown in Figure 4, the mRNA expression levels of SOD1, SOD2, CAT, and GSH in the skin tissues of mice in the UVB group were significantly reduced compared with those in the normal group. Compared with the UVB group, the mRNA expression of SOD1, SOD2, CAT, and GSH in the skin and liver of the VC + UVB group, the clove-L + UVB group, and the clove-H + UVB group all increased to different degrees, and the mRNA expression levels of the above indexes in the clove-L + UVB group were similar to those in the normal group.

**Figure 5:**
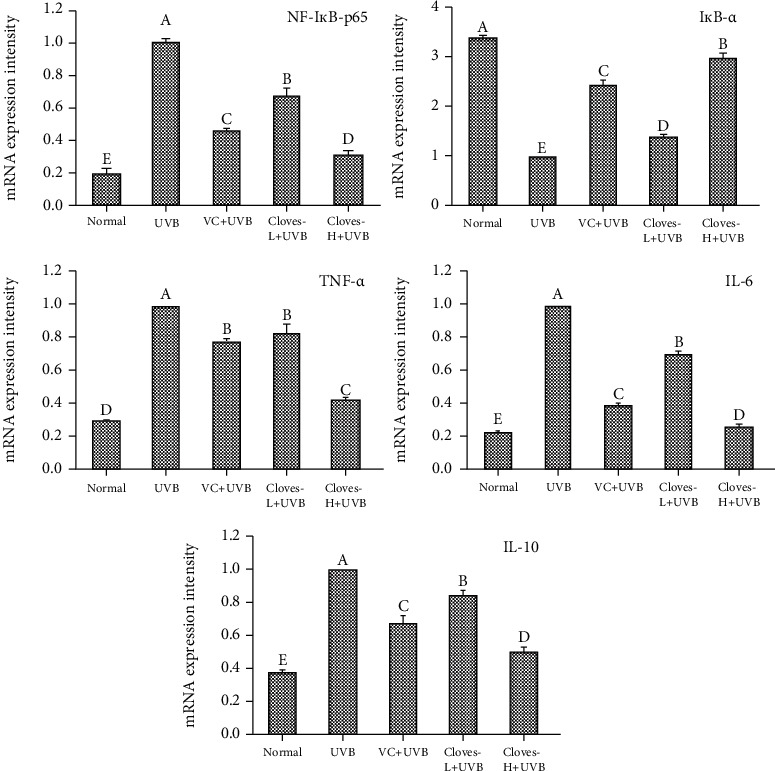
mRNA expression levels of NF-*κ*B p65, I*κ*B-*α*, TNF-*α*, IL-6, and IL-10 in the skin tissue of mice. ^a–e^Different letters indicate significant differences between the two groups (*P* < 0.05) according to Duncan's honestly significantly different test.

As shown in Figure 5, compared with the normal group, UVB irradiation increased the mRNA expression level of NF-*κ*B p65 in the skin tissues of mice, decreased the expression of NF-*κ*B p65 inhibitor gene I*κ*B-*α*, and also downregulated the expression of IL-10, which is related to the NF-*κ*B p65 signaling pathway. The expression of TNF-*α* and IL-6 was upregulated, especially in the UVB group. Compared with the UVB group, the mRNA expression of I*κ*B-*α* and IL-10 in the skin and liver of the VC + UVB, clove-L + UVB, and clove-H + UVB groups was increased to different degrees, while the expression of NF-*κ*B p65, TNF-*α*, and IL-6 was decreased. The mRNA expression levels of the above indexes in the clove-H + UVB group were similar to those in the normal group. These results suggest that cloves can effectively inhibit the activation of the NF-*κ*B p65 signaling pathway by upregulating the expression of I*κ*B-*α* in skin and liver tissues.

**Figure 6:**
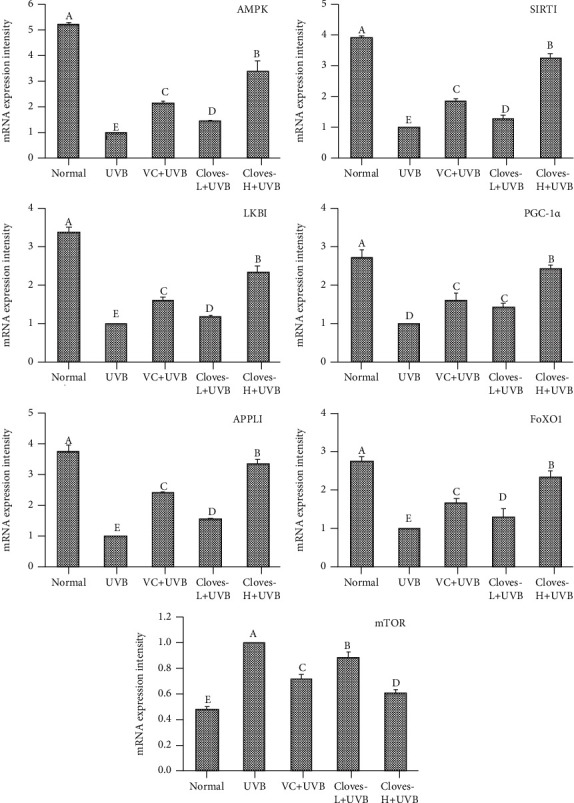
mRNA expression levels of AMPK, SIRT1, mTOR, LKB1, PGC-1*α*, APPL1, and FoxO1 in the skin tissue of mice. ^a–e^Different letters indicate significant differences between the two groups (*P* < 0.05) according to Duncan's honestly significantly different test.

Figure 6 shows that the mRNA expression of AMPK, SIRT1, LKB1, PGC-1*α*, APPL1, and FoxO1 in the skin tissues of mice in the normal group was the highest, and the expression of mTOR was the lowest. However, the expression levels of the above indicators in the skin and liver of mice in the UVB group exhibited a completely opposite trend compared with the normal group. There were significant differences between the two, indicating that UVB irradiation caused energy metabolism disorders in mice and increased the oxidative stress response and inflammatory response. After the VC and clove treatment, the mRNA expression of AMPK in the skin and liver was increased, and the mRNA expression of SIRT1, LKB1, PGC-1*α*, APPL1, and FoxO1, which are related to the AMPK signaling pathway, was also increased, while the expression of mTOR was decreased. The expression levels of the above indexes in the clove-H + UVB group were similar to those in the normal group, indicating that cloves can effectively promote cell energy synthesis by regulating the AMPK signaling pathway and thereby reduce the damage caused by oxidative stress and inflammation.

## 6. Discussion

An oxidation-antioxidation imbalance in the body is closely related to the occurrence and development of oxidative stress injury. Long-term UVB irradiation will induce a severe oxidative stress response, thus accelerating skin injury [18]. Superoxide dismutase (SOD) is the only antioxidant enzyme capable of scavenging superoxide anion free radicals (O_2_·^−^) in organisms. The level of SOD activity is closely related to aging and generally decreases with age [19]. Malondialdehyde (MDA) is the end product of metabolism produced by the lipid peroxidation chain reaction induced by free radicals. It is also an extremely active agent that is able to crosslink the dermal structure with macromolecules, resulting in skin with age spots, decreased softness, and increased wrinkles. With increased accumulation of free radicals, the greater the amount of MDA that is produced, the greater the damage to the body [20].

Catalase (CAT) is an oxidoreductase, most of which are heme catalases, and they play an important role in biosynthesis, degradation, and defense [21]. Hydrogen peroxide (H_2_O_2_) is an intermediate product of reactive oxygen species. If it is excessively accumulated in the body, it will lead to an imbalance in the antioxidant system that will further increase the oxidative stress response and cause cell damage and apoptosis [22]. Glutathione (GSH) is an important antioxidant that scavenges free radicals in the body. Because GSH is easily oxidized by some substances, it can protect the sulfhydryl groups in many proteins and enzymes from being oxidized by harmful substances in vivo, so as to ensure the normal performance of physiological functions of proteins, enzymes, and other molecules [23]. Cloves maintain T-SOD, CAT, MDA, and H_2_O_2_ in the mouse skin tissue at relatively normal levels, but they can also greatly increase the serum T-SOD and CAT levels. In addition, cloves upregulate the mRNA expression of SOD1, SOD2, CAT, and GSH in skin and liver tissues at the gene level. This indicates that cloves can decrease the UVB-induced skin damage in mice by regulating the oxidative stress levels of the body and can also increase the overall antioxidant level of the body from serum and liver levels, with a greater effect than that of vitamin C.

NAD^+^ and Na^+^-K^+^-ATPase are enzymes that react very sensitively to skin damage. Studies have shown that UVB irradiation can cause rapid reduction of NAD^+^ and Na^+^-K^+^-ATPase in skin tissues [24]. As a precursor of NAD^+^ synthesis, orally consumed cloves can increase the level of NAD^+^ in skin tissues, which will further increase the level of Na^+^-K^+^-ATPase, and ultimately prevent the damage caused by UVB irradiation. The regulation of Na^+^-K^+^-ATPase activity results in cells with necessary vitality, and any decrease in its activity will lead to obstacles in intracellular energy production and ion transport, thus affecting cell function and accelerating cell damage [25]. In this study, it was found that the levels of NAD^+^ and Na^+^-K^+^-ATPase in the skin tissues of mice in the UVB group were significantly lower than those in the normal group, indicating that UVB irradiation caused skin energy metabolism disorders. The levels of NAD^+^ and Na^+^-K^+^-ATPase in the skin of mice treated with cloves were significantly increased and were close to those in the normal group.

When the skin is exposed to UV light for a sufficient amount of time, Na^+^-K^+^-ATPase will decrease, leading to abnormal osmotic pressure inside and outside the skin cells, destruction of the integrity and fluidity of the cell membrane, and further inflammatory reactions such as dryness, itching, erythema, and edema [26]. NF-*κ*B is an important transcription factor that participates in the immune response of the body. Under normal conditions, NF-*κ*B binds to its inhibitory protein I*κ*B in an inactive manner. Studies have shown that abnormal stimulation in the body accelerates the phosphorylation of I*κ*B protein, which activates NF-*κ*B to further increase the release of proinflammatory cytokines such as TNF-*α*, IL-6, IL-12, COX-2, and iNOS [27, 28]. Many studies have shown that the NF-*κ*B signaling pathway is closely related to the inflammatory response in the body, and NF-*κ*B signaling dysfunction is often seen during skin injury [29, 30].

I*κ*B-*α* is a repressor of NF-*κ*B, and it can mask the nuclear localization signal of NF-*κ*B and thus enables NF-*κ*B to exist in cells in the form of an inactive complex. Almost all NF-*κ*B inducers have been shown to rapidly activate NF-*κ*B due to degradation of I*κ*B-*α*, and thus the activation of NF-*κ*B can be prevented by blocking the phosphorylation of I*κ*B-*α* [31]. By measuring the levels of IL-4, IL-6, IL-10, TNF-*α*, and IL-1*β* in the serum and skin of mice, it was observed that cloves regulated the level of inflammatory injury in the body. The possible reason for this is that cloves affect the level of Na^+^-K^+^-ATPase, thereby regulating the level of inflammation in the body.

AMP-activated protein kinase (AMPK) is a highly conserved threonine kinase that is widely found in various eukaryotic cells and plays an important role in maintaining the balance of intracellular energy metabolism. Once activated, AMPK mainly regulates four metabolic categories in mammals: protein metabolism, lipid metabolism, carbohydrate metabolism, and autophagy and mitochondrial homeostasis, covering almost all physiological metabolic activities of living organisms. AMPK is also a key protein involved in multiple signaling pathways that block the metabolism of rapidly proliferating tumors and restore normal function to the liver and other tissues of diabetics [32].

The AMPK signaling pathway has been widely reported in host defense, such as infection, oxidative stress, inflammation, and immunity. In young cells, high levels of AMPK promote the activity of SIRT1, FoxO1, and PGC-1*α*, and inhibit the activity of NF-*κ*B [33–35]. The endoplasmic reticulum and oxidative stress are effective inducers of NF-*κ*B signal transduction, while AMPK protects cells from mitochondrial dysfunction and inhibits endoplasmic reticulum and oxidative stress. NF-*κ*B signaling is enhanced with decreased AMPK activity after cell aging [36].

The related genes also involved in AMPK signaling are Appl1, LKB1, SIRT1, mTOR, FoxO1, peroxisome proliferating receptor *γ* coactivator *α* (PGC-1*α*), etc. [37]. APPL1 is a functional protein located on chromosome 3, composed of 709 amino acids, and originates from a gene upstream of AMPK. It can mediate a variety of cell signal transductions, play a role in numerous cell responses, and regulate the cellular inflammatory response, antioxidation, and arteriosclerosis. LKB1 is an upstream kinase of AMPK and is mainly distributed in the nucleus. It activates AMPK by directly phosphorylating threonine 172 on the *α*-subunit of AMPK, thereby regulating cellular energy metabolism [38]. SIRT1, a member of the sirtuin family, is an important nicotinamide adenine dinucleotide- (NAD^+^-) dependent deacetylase that plays an important regulatory role in energy metabolism, aging, and other functions. SIRT1 enhances the expression of PGC-1*α* through deacetylation, which, as an important regulator of mitochondrial biosynthesis, directly regulates mitochondrial number and function [39].

mTOR is an evolutionarily conserved serine/threonine protein kinase that acts as an integrator of growth factors and nutritional signaling. Activation of the mTOR pathway is thought to be closely related to the pathogenesis of cutaneous melanoma, while AMPK activation inhibits mTOR and its effectors, thereby reducing energy consumption [40]. FoxO1 is a member of the Foxhead family and is mainly involved in the regulation of various physiological and biochemical processes such as oxidative stress, cycle arrest, autophagy, and metabolism, as well as various genes involved in cell metabolism related to cell death and oxidative stress responses [41]. As a regulatory factor of AMPK, PGC-1*α* participates in mitochondrial biosynthesis, increases mitochondrial respiration and oxidative capacity, and regulates fatty acid oxidation by regulating adaptive thermogenesis, glycolipid metabolism, and blood glucose balance. PGC-1*α* regulates adaptive thermogenesis, glycolipid metabolism, and blood glucose balance, participates in mitochondrial biosynthesis, and increases the mitochondrial respiration and oxidative capacity [42].

Once activated, AMPK stimulates the catabolic pathways that absorb nutrients and produce ATP, while inhibiting the anabolic pathways that consume ATP, thereby assisting in the maintenance of cellular energy balance in the face of energy deficiency. Up to 20-25% of the total body consumption of ATP is caused by reactions catalyzed by Na^+^-K^+^-ATPase, which may be an important factor that influences AMPK pathway regulation [43]. In this study, we determined that cloves also affect the mRNA expression of SIRT1, LKB1, PGC-1*α*, APPL1, FoxO1, and mTOR in the AMPK pathway by regulating Na^+^-K^+^-ATPase, which can effectively promote cell energy synthesis by regulating the AMPK signaling pathway. This can protect the skin by reducing the damage caused by oxidative stress and inflammation.

Rutin, isoquercitrin, ferulic acid, dihydroquercetin, and quercitrin are all excellent antioxidant compounds [44–48]. Our study also showed that ferulic acid exhibited antibacterial effects [49], and quercitrin exhibited antiviral effects [50]. It has also been proven that rutin has a very strong ability to scavenge free radicals [51]. Rutin can regulate Na^+^-K^+^-ATPase, which thus affects the oxidative stress levels in animals [52]. Moreover, rutin has a strong absorption effect on UV radiation. As a natural sunscreen, the absorption rate of UV radiation is as high as 98% when 10% rutin is added [53]. Cloves contain all five compounds, which help protect the skin.

## 7. Conclusions

According to the experimental results of this study, cloves can normalize the levels of SOD, CAT, GSH, MDA, and H_2_O_2_ oxidative stress indexes in serum and skin and reduce the inflammatory damage regulated by the NF-*κ*B signaling pathway. Cloves also regulate the energy metabolism maintained by the AMPK signaling pathway. Cloves can decrease UVB damage through their influence on Na^+^-K^+^-ATPase, which leads to a reduction in oxidation and inflammation in mice, thereby inhibiting skin injury and protecting the skin. The five active antioxidant compounds in cloves (rutin, isoquercitrin, ferulic acid, dihydroquercetin, and quercitrin) contribute to the prevention of UVB skin damage, and therefore, cloves serve as an important nutrient to protect the skin. In addition, this study is limited to animal experiments in vivo, and the effect on human body needs further clinical experiments.

## Figures and Tables

**Table 1:** Mobile phase conditions of HPLC in this study.

Time (min)	Current speed (mL/min)	0.5% acetic acid (%)	Acetonitrile (%)
0	0.5	88	12
30	0.5	55	45
35	0.5	0	100
40	0.5	0	100

**Table 2:** Sequences of the primers used for the mice skin tissues.

Gene	Sequences
*NF-κB p65*	F: 5′-GAGGCACGAGGCTCCTTTTCT-3′
	R: 5′-GTAGCTGCATGGAGACTCGAACA-3′
*IκB-α*	F: 5′-TGAAGGACGAGGAGTACGAGC-3′
	R: 5′-TGCAGGAACGAGTCTCCGT-3′
*TNF-α*	F: 5′-CAGGCGGTGCCTATGTCTC-3′
	R: 5′-GCTGCAACAGGGGGTAACAT-3′
*IL-6*	F: 5′-CTGCAAGAGACTTCCATCCAG-3′
	R: 5′-AGTGGTATAGACAGGTCTGTTGG-3′
*IL-10*	F: 5′-CTTACTGACTGGCATGAGGATCA-3′
	R: 5′-GCAGCTCTAGGAGCATGTGG-3′
*SOD1*	F: 5′-AACCAGTTGTGTTGTCAGGAC-3′
	R: 5′-CCACCATGTTTCTTAGAGTGAGG-3′
*SOD2*	F: 5′-CAGACCTGCCTTACGACTATGG-3′
	R: 5′-CTCGGTGGCGTTGAGATTGTT-3′
*CAT*	F: 5′-GGAGGCGGGAACCCAATAG-3′
	R: 5′-GTGTGCCATCTCGTCAGTGAA-3′
*GSH*	F: 5′-CCACCGTGTATGCCTTCTCC-3′
	R: 5′-AGAGAGACGCGACATTCTCAAT-3′
*AMPK*	F: 5′-GTCAAAGCCGACCCAATGATA-3′
	R: 5′-CGTACACGCAAATAATAGGGGTT-3′
*LKB1*	F: 5′-CTGGACTCCGAGACCTTATGC-3′
	R: 5′-CAAGCTGGATCACATTCCGAT-3′
*SIRT1*	F: 5′-TGATTGGCACCGATCCTCG-3′
	R: 5′-CCACAGCGTCATATCATCCAG-3′
*mTOR*	F: 5′-CAGTTCGCCAGTGGACTGAAG-3′
	R: 5′-GCTGGTCATAGAAGCGAGTAGAC-3′
*PGC-1α*	F: 5′-TATGGAGTGACATAGAGTGTGCT-3′
	R: 5′-GTCGCTACACCACTTCAATCC-3′
*APPL1*	F: 5′-AGCCAGTGACCCTTTATATCTGC-3′
	R: 5′-AGGTATCCAGCCTTTCGGGTT-3′
*FoxO1*	F: 5′-CCCAGGCCGGAGTTTAACC-3′
	R: 5′-GTTGCTCATAAAGTCGGTGCT-3′
*β-Actin*	F: 5′-CATGTACGTTGCTATCCAGGC-3′
	R: 5′-CTCCTTAATGTCACGCACGAT-3′

**Table 3:** T-SOD, CAT activities and H_2_O_2_, and AGE levels in the serum and skin tissue of mice.

Group	T-SOD (U/mL)	CAT (U/mL)	H_2_O_2_ (mmol/L)	AGEs (pg/mL)
Normal	147.56 ± 14.85^a^	45.80 ± 5.11^a^	12.07 ± 1.56^e^	45.83 ± 3.65^e^
UVB	58.87 ± 8.17^e^	14.59 ± 2.19^e^	68.58 ± 4.78^a^	167.83 ± 8.71^a^
VC + UVB	105.22 ± 10.17^c^	28.91 ± 3.02^c^	31.03 ± 3.87^c^	89.78 ± 6.67^c^
Cloves-L + UVB	82.06 ± 8.93^d^	21.07 ± 2.65^d^	48.68 ± 4.33^b^	125.17 ± 7.02^b^
Cloves-H + UVB	120.09 ± 11.22^b^	33.07 ± 3.15^b^	18.49 ± 3.17^d^	67.82 ± 5.14^d^
Group	T-SOD (U/mgprot)	CAT (U/mgprot)	H_2_O_2_ (mmol/gprot)	AGEs (pg/mL)
Normal	40.69 ± 3.11^a^	26.77 ± 3.89^a^	4.57 ± 0.32^e^	112.08 ± 5.69^e^
UVB	6.88 ± 0.47^e^	10.82 ± 2.07^d^	18.88 ± 1.03^a^	378.83 ± 8.91^a^
VC + UVB	22.72 ± 4.52^c^	18.04 ± 1.95^b^	7.43 ± 0.44^c^	220.65 ± 8.36^c^
Cloves-L + UVB	10.59 ± 0.55^d^	14.13 ± 1.86^c^	10.71 ± 0.65^b^	280.75 ± 9.18^b^
Cloves-H + UVB	30.99 ± 4.36^b^	21.84 ± 2.01^ab^	6.02 ± 0.28^d^	169.69 ± 11.25^d^

^a–e^Different letters indicate significant differences between the two groups (*P* < 0.05) according to Duncan's honestly significantly different test.

**Table 4:** TNF-*α*, IL-1*β*, IL-4, IL-6, and IL-10 levels in the serum and skin tissue of mice.

Group	TNF-*α* (ng/L)	IL-1*β* (ng/L)	IL-4 (pg/L)	IL-6 (pg/mL)	IL-10 (pg/mL)
Normal	125.92 ± 12.36^e^	9.74 ± 0.71^e^	116.85 ± 12.57^a^	14.50 ± 2.15^e^	384.75 ± 21.59^a^
UVB	615.26 ± 22.57^a^	23.66 ± 1.18^a^	63.08 ± 8.52^b^	81.36 ± 5.97^a^	148.25 ± 18.97^e^
VC + UVB	351.08 ± 18.93^c^	14.25 ± 0.59^c^	89.53 ± 7.32^ab^	38.79 ± 4.89^c^	268.75 ± 23.91^c^
Cloves-L + UVB	587.59 ± 16.77^b^	18.75 ± 1.02^b^	75.85 ± 7.62^b^	64.12 ± 6.03^b^	211.53 ± 20.55^d^
Cloves-H + UVB	241.85 ± 20.55^d^	11.28 ± 0.63^d^	102.56 ± 8.92^a^	24.18 ± 4.71^d^	310.53 ± 16.78^b^
Group	TNF-*α* (ng/mgprot)	IL-1*β* (ng/mgprot)	IL-4 (ng/mgprot)	IL-6 (ng/mgprot)	IL-10 (ng/mgprot)
Normal	74.56 ± 4.77^e^	5.03 ± 0.47^e^	144.40 ± 10.16^a^	30.85 ± 3.89^d^	587.27 ± 32.05^a^
UVB	168.92 ± 7.12^a^	16.42 ± 0.77^a^	58.97 ± 6.15^d^	70.86 ± 4.47^a^	178.06 ± 22.56^e^
VC + UVB	92.88 ± 5.53^c^	10.89 ± 0.49^c^	109.25 ± 8.72^b^	51.09 ± 4.25^b^	407.83 ± 25.11^c^
Cloves-L + UVB	125.05 ± 6.62^b^	13.27 ± 0.69^b^	88.33 ± 7.02^c^	58.75 ± 3.35^b^	320.47 ± 26.32^d^
Cloves-H + UVB	79.83 ± 6.03^d^	7.32 ± 0.88^d^	135.09 ± 9.44^a^	44.32 ± 4.06^c^	510.88 ± 20.69^b^

^a–e^Different letters indicate significant differences between the two groups (*P* < 0.05) according to Duncan's honestly significantly different test.

## Data Availability

The datasets generated for this study are available upon request to the corresponding author.

## Abbreviations

UVB: Ultraviolet B
H&E: Hematoxylin-eosin staining
qPCR: Quantitative polymerase chain reaction
HPLC: High performance liquid chromatography
T-SOD: Total superoxide dismutase
CAT: Catalase
AGEs: Advanced glycation endproducts
TNF-*α*: Tumor necrosis factor alpha
IL-1*β*: Interleukin-1 beta
IL-4: Interleukin-4
IL-6: Interleukin-6
IL-10: Interleukin-10
SOD1: Cu/Zn superoxide dismutase
SOD2: Mn superoxide dismutase
GSH: Glutathione
NF-*κ*B p65: Nuclear factor kappa-B p65
I*κ*B-*α*: Inhibitor kappa B alpha
AMPK: Adenosine 5′-monophosphate- (AMP-) activated protein kinase
LKB1: Liver kinase B1
PGC-1*α*: Peroxlsome proliferator-activated receptor-*γ* coactlvator-1 alpha
APPL1: Adaptor protein containing pH domain, PTB domain, and leucine zipper motif 1
FoxO1: Forkhead transcription factor O1
mTOR: Target of rapamycin
ATP: Adenosine triphosphate.
